# Prognostic nomogram for acute pancreatitis after percutaneous biliary stent insertion in patients with malignant obstruction

**DOI:** 10.1186/s12876-022-02554-w

**Published:** 2022-11-07

**Authors:** Chen Xu, Yiming Gu, Weizhong Zhou, Guoxiong Xu, Sheng Liu, Haibin Shi

**Affiliations:** 1 Department of Intervention Radiology, Suzhou Municipal Hospital Affiliated to Nanjing Medical University, 242 Guangji Road, Gusu District, Suzhou, 215008 China; 2 Department of Interventional Radiology, The First Affiliated Hospital of Nanjing Medical University, 300 Guangzhou Road, Gulou District, Nanjing, 210029 China

**Keywords:** Pancreatitis, Nomogram, Percutaneous transhepatic biliary stent insertion, Malignant biliary obstruction

## Abstract

**Objective:**

This study aimed to develop and validate a nomogram to predict the risk of pancreatitis after percutaneous transhepatic biliary stent insertion (PTBS) in patients with malignant biliary obstruction (MBO).

**Materials and methods:**

We enrolled 314 patients who underwent PTBS for MBO from March 2016 to July 2021 in this retrospective study. We used univariate analysis to identify potential risk factors, while a multivariate logistic regression model was employed to establish a nomogram for predicting the risk of pancreatitis. The discrimination and calibration of the nomogram were evaluated by estimating the area under the receiver operator characteristic curve (AUC) and by bootstrap resampling and visual inspection of the calibration curve. The clinical utility of the nomogram was assessed using decision curve analysis (DCA).

**Results:**

After the procedure, 41 (13.1%) patients developed pancreatitis. Based on multivariate logistic regression analysis, young age (*OR* = *2.57, 95% CI 1.16 to 5.69*), stent insertion across the papilla (*OR* = *6.47, 95% CI 2.66 to 15.70*), and visualization of the pancreatic duct (*OR* = *15.40, 95% CI 6.07 to 39.03*) were associated with an elevated risk of pancreatitis. Importantly, the performance of the nomogram was satisfactory, with an identical AUC (*0.807, 95% CI 0.730 to 0.883*) and high-level agreement between predicted and observed probabilities as suggested in calibration curves. The DCA curve subsequently confirmed the clinical utility.

**Conclusion:**

A predictive nomogram for pancreatitis after PTBS in patients with MBO was successfully established in the present study.

## Introduction

Malignant biliary obstruction (MBO) can be caused by pancreatic cancer, hepatic cancer, gallbladder carcinoma, or other malignant tumors. Patients are often diagnosed at an advanced stage when tumor resection is impossible [1]. Generally, a stent can be placed endoscopically or percutaneously to alleviate the clinical symptoms and improve living quality. Endoscopic biliary stenting (EBS) is the mainstay for MBO and is recommended for most patients. Percutaneous transhepatic biliary stent insertion (PTBS) is an alternative option when EBS is failed and has its own advantages [2–6].

The complications of PTBS include cholangitis, bleeding, and perforation, among others, of which pancreatitis is a serious complication that requires further investigation [7–9]. Studies have shown that mild pancreatitis can sometimes progress to be severe and even fatal after the procedure [10, 11]. However, although several studies reported on the risk factors related to pancreatitis after PTBS, their conclusions differed from one another [1, 6, 12]. More importantly, there is a lack of an effective and simple model to predict the risk of post-procedural pancreatitis so as to provide timely treatment and prevent negative outcomes.

The goal of this study was to develop and validate a nomogram that incorporated various patient and procedure characteristics to predict the risk of pancreatitis after PTBS in patients with MBO.

## Materials and methods

### Patients

**Fig. 1:**
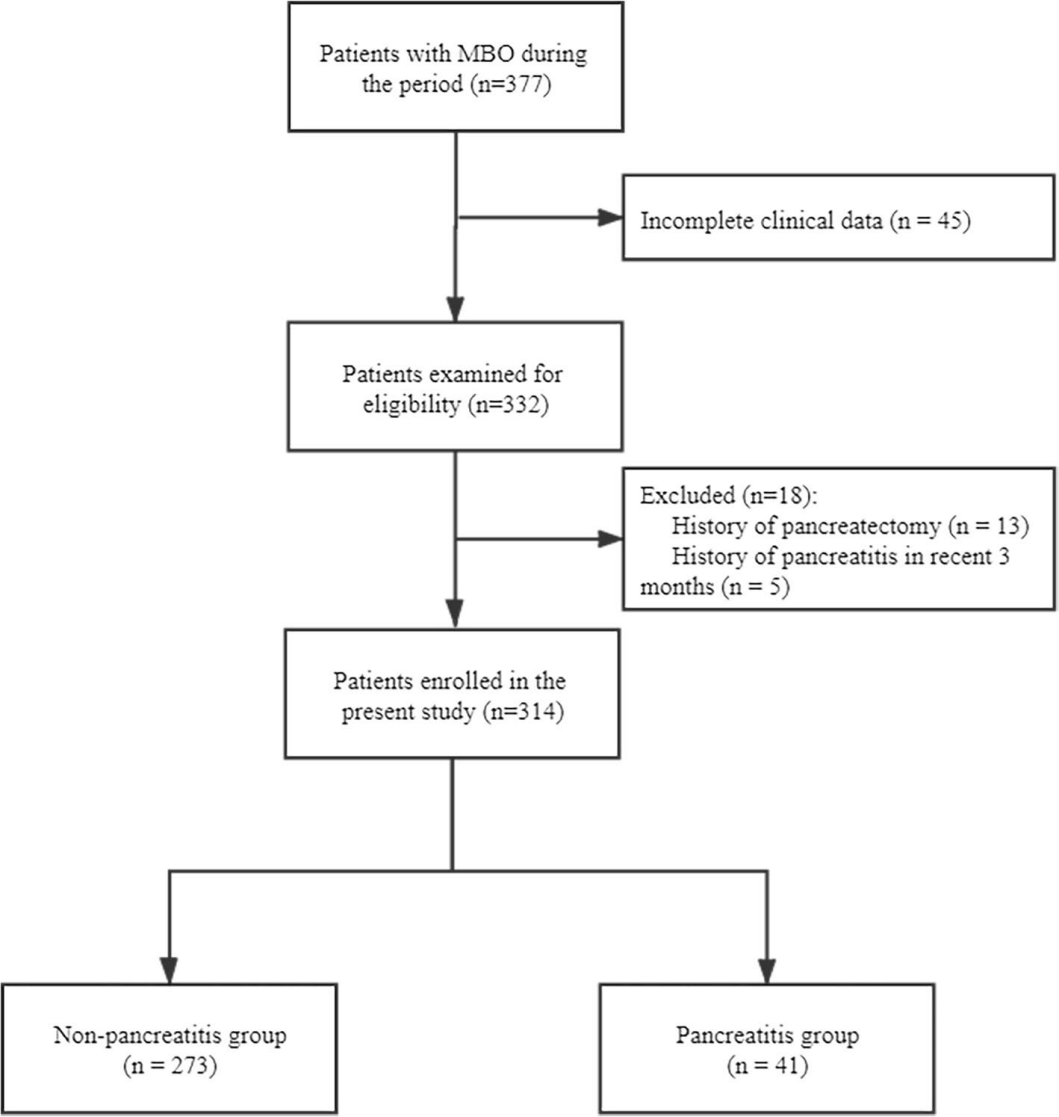
Trial profile

This retrospective study was approved by the institutional review board of our hospital. Data collection consent was obtained. Data of 377 consecutive patients with MBO who underwent PTBS in our center between March 2016 and July 2021 were collected. For this study, inclusion criteria included: (1) complete clinical data including laboratory indexes and imaging information; (2) MBO confirmed based on radiological and/or pathological findings. Exclusion criteria included: (1) patients with a history of pancreatitis in the recent 3 months at admission or (2) a history of pancreatectomy. After that, 314 patients were included in this study. Among them, 159 were men and 155 were women. The median age of the patients was 65.0 years (range, 28—92 years). The patient inclusion process is illustrated in Fig. 1. The diagnosis of the primary tumor was established based on laboratory and radiology findings of 145 patients and pathological results of 169 patients.

### Stent insertion

All patients were required to fast at least 8 h prior to the procedure. The stent insertion was performed under local anesthesia. The intrahepatic bile duct was punctured using a 21-gauge Chiba needle (Cook, Bloomington, IN, USA). In case of a successful puncture, a 0.018-inch guidewire was inserted, and thereafter a 4F introducer sheath (Neff Percutaneous Access Set, Cook, Bloomington, IN, USA) was introduced. We then performed cholangiography to evaluate the obstruction site. Following this, a 0.035-inch guidewire was advanced to the duodenum across the obstruction site with a 4F catheter. After measurement of the length of stricture, the stent was introduced over the guidewire and then deployed across the stricture to cover the bile duct approximately 1.5—2 cm distal and proximal to the obstruction to prevent tumor infiltration. Stent graft patency was confirmed with repeat cholangiography. Moreover, the external drainage tube (8F, Cook, Bloomington, IN, USA) was inserted in patients with infection, and the iodine-125 seeds (0.8 mci, Xinke, Shanghai, China) strand was inserted for intraluminal radiotherapy in some patients with their permission. The puncture approach was occluded with gel foam pledgets through a sheath. Three types of uncovered SEMS (Self-Expanding Metallic Stent) with a diameter of 8 mm and lengths from 60 to 100 mm were used in the current study (E-Luminexx [Bard Peripheral Vascular, Tempe, AZ], S.M.A.R.T [Cordis, Milpitas, CA], and Zilver [Cook, Bloomington, IN]). All procedures were carried out by two interventional radiologists with more than 10 years of experience.

### Definition and follow-up

Acute pancreatitis was diagnosed based on the Atlanta classification [13], which requires the presence of two or more of the following criteria: persistent abdominal pain accompanied by vomiting and nausea; the level of serum amylase of at least three times over the limit of normal; and radiology features including CT or ultrasonography.

During the period of hospitalization, all patients were followed daily monitoring the levels of serum amylase and clinical condition. The first postoperative serum amylase levels were determined at 3 h after the procedure. Radiology examinations were used to confirm pancreatitis when the serum amylase levels were over three times upper the limit of normal without clinical symptoms. Patients with pancreatitis received somatostatin and fasting therapy.

### Data collection

Data of each patient was extracted from individual medical records and image systems. The characteristics of patients consisted of age and gender, primary tumor, underlying disease, previous biliary drainage, preoperative infection, laboratory indices, hs-CRP, and location of stricture. The procedure-related characteristics included operation time, external drainage tube insertion, Iodine-125 seed strand insertion, stent length, number of stents, stent insertion across the papilla, and visualization of the pancreatic duct.

### Statistical analysis

For subgroup analysis, enrolled patients were classified into two groups (non-pancreatitis and pancreatitis) according to clinical outcomes. Missing data were excluded from the analyses. Continuous variables were evaluated using the Mann–Whitney U-test and depicted as medians and IQR (Inter-Quartile Range). Categorical data were compared using chi-square or Fisher's exact tests as appropriate and presented as frequencies.

We developed a nomogram for pancreatitis after PTBS in three steps:(1) a univariate analysis with one variable was used at a time to identify potential risk factors(ie, young age, stent insertion across the papilla, and visualization of the pancreatic duct); (2) significant variables above were subjected to the backward multivariate logistic regression analysis method to establish the independent predictor for post-procedural pancreatitis; (3) a nomogram was developed by entering the results of regression into the “rms” and “shiny” package of R software. For developing a nomogram, each factor was scored on basis of estimated logistic regression coefficients. The biggest impact factor was determined and sequentially other factors were scored in proportion to the points assigned to the biggest impact factor. This translated complex mathematical models into a simple graph of scaled variables facilitating a quick approximation of event probability [14].

We further evaluated the performance of the nomogram in terms of discrimination and calibration. The ability of the nomogram to distinguish non-pancreatitis from pancreatitis was assessed by calculating the area under the receiver operator characteristic curve (AUC). Moreover, the predictive performance was validated with bootstrap resampling repeated 1000 times and then compared the predicted and observed probabilities of pancreatitis in patients with MBO. The decision curve analysis (DCA) was used to confirm the clinical utility of this predictive scoring system. A *P*-value of < 0.05 was considered statistically significant. All data analyses were implemented using R software V.4.0.2 (Beijing Foreign Studies University, Beijing, China; www.r-project.org).

## Results

### Predictor variables and complications

Among the 314 patients, 41 (13.1%) developed pancreatitis. All patients recovered at a mean of 3.2 days (range, 1–7 days) under somatostatin and fasting therapy during the period of hospitalization. No severe pancreatitis occurred and none needed further surgical therapy or intensive care. The baseline of patients was summarized in Table 1. Patients with stent insertion across the papilla represented the larger proportion of pancreatitis (80.5%), with only 19.5% of pancreatitis patients with stent above the papilla. Visualization of the pancreatic duct was more common among pancreatitis than among non-pancreatitis (46.3% vs 5.1%), and young patients (< 60 years) were more common as well (56.1% vs 34.1%). There were no significant differences in other factors between the two groups (Table 1). Subsequently, the variables of young age (*p* = 0.003), stent insertion across the papilla (*p* < 0.001), and visualization of the pancreatic duct (*p* < *0.001*) in the univariate analysis were considered for the multivariate logistic regression analysis. Finally, the results of backward stepwise regression analysis revealed that the following factors were markedly associated with increased risk of pancreatitis after PTBS: young age (*OR* = *2.57, 95% CI 1.16 to 5.69*), stent insertion across the papilla (*OR* = *6.47, 95% CI 2.66 to 15.70*), and visualization of the pancreatic duct (*OR* = *15.40, 95% CI 6.07 to 39.03*) (Table 2). Besides, fifty-one (16.2%) patients with other complications were observed in this study, including 29 (9.2%) of puncture site pain, 11 (3.5%) of infection, 8 cases (2.5%) of bile leak, and 3 (1.0%) of bleeding. All patients recovered under conservative treatment before discharge.

**Table 1:** Univariate analysis of the risk factors for acute pancreatitis after PTBS

	All patients	Groups			
Variables	(*n* = 314)	Non-AP (*n* = 273)	AP (*n* = 41)	*Z / χ2*	*p-value*
**Patient characteristics, frequency (%)**					
Gender				0.856	0.355
Male	159 (50.6)	141 (51.6)	18 (43.9)		
Female	155 (49.4)	132 (48.4)	23 (56.1)		
Age				8.829	0.003
≥ 60 years	196 (62.4)	179 (65.6)	17 (41.5)		
< 60 years	118 (37.6)	94 (34.4)	24 (58.5)		
Primary tumor				0.509	0.476
Pancreatic cancer	46 (14.6)	42 (15.4)	4 (15.0)		
Non-Pancreatic cancer	268 (85.4)	231 (84.6)	37 (85.0)		
Hypertension				0.231	0.630
Yes	112 (35.7)	96 (35.2)	16 (39.0)		
No	202 (64.3)	177 (64.8)	25 (61.0)		
Diabetes mellitus				2.764	0.096
Yes	49 (15.6)	39 (14.3)	10 (24.4)		
No	265 (84.4)	234 (85.7)	31 (75.6)		
Cardiovascular disease				0.652	0.419
Yes	30 (9.6)	28 (10.3)	2 (4.9)		
No	284 (90.4)	245 (89.7)	39 (85.1)		
Previous biliary drainage				0.067	0.795
Yes	132 (42.0)	114 (41.8)	18 (43.9)		
No	182 (58.0)	159 (58.2)	23 (56.1)		
Preoperative infection				0.994	0.319
Yes	94 (29.9)	79 (28.9)	15 (36.6)		
No	220 (70.1)	194 (71.1)	26 (63.4)		
Location of stricture				3.542	0.170
Upper bile duct	92 (29.3)	85 (31.1)	7 (17.1)		
Lower bile duct	151 (48.1)	127 (46.5)	24 (58.5)		
Whole bile duct	71 (22.6)	61 (22.4)	10 (24.4)		
Laboratory indices, median (IQR)					
ALT (U/L)	73.3 (42.4–124.5)	72.9 (42.5–127.0)	77.3 (42.7–121.8)	-0.183	0.855
AST (U/L)	89.6 (56.4–145.9)	87.8 (56.1–147.0)	98.8 (63.9–159.2)	-0.602	0.547
ALP (U/L)	420.3 (255.0–701.4)	414.6 (270.3–684.3)	491.0 (208.9–808.9)	-0.123	0.902
TBIL (μmol/L)	197.5 (111.8–318.9)	192.5 (115.6–320.9)	203.2 (94.6–291.4)	-0.306	0.759
DBIL (μmol/L)	152.2 (94.0–240.8)	156.0 (94.3–245.7)	145.5 (80.6–232.1)	-0.468	0.639
hs-CRP, mg/L				2.092	0.148
≥ 8	108	98 (35.9)	10 (24.4)		
< 8	206	175 (64.1)	31 (75.6)		
**Procedure characteristics, frequency (%)**					
Operation time				0.128	0.721
≥ 60 min	68 (21.7)	60 (22.0)	8 (19.5)		
< 60 min	246 (78.3)	213 (78.0)	33 (80.5)		
External drainage tube				0.011	0.918
Yes	67 (21.3)	58 (21.2)	9 (22.0)		
No	247 (78.7)	215 (78.8)	32 (78.0)		
Iodine-125 seed strand				1.208	0.272
Yes	64 (20.4)	53 (19.4)	11 (26.8)		
No	250 (79.6)	220 (80.6)	30 (73.2)		
Stent length				2.890	0.236
6 cm	119 (37.9)	106 (38.8)	13 (31.7)		
8 cm	158 (50.3)	136 (50.6)	20 (48.8)		
10 cm	37 (11.8)	31 (10.6)	8 (19.5)		
Number of stents				1.044	0.307
One	196 (62.4)	182 (66.7)	24 (58.5)		
Multiple	118 (37.6)	91 (33.3)	17 (41.5)		
Stent placement across the papilla				29.150	< 0.001
Yes	131 (41.7)	98 (35.9)	33 (80.5)		
No	183 (58.3)	175 (64.1)	8 (19.5)		
Visualization of the pancreatic duct				64.377	< 0.001
Yes	33 (10.5)	14 (5.1)	19 (46.3)		
No	281 (89.5)	259 (94.9)	22 (53.7)		

^*^ Operation time was defined as the time from successful puncture to repeat cholangiography confirmed with stent patency

^*^ Stent length was evaluated as the maximum length while multiple-stent insertion

^*^ *IQR* Inter-quartile range, *ALT* Alanine aminotransferase, *AST* Aspartate aminotransferase, *ALP* Alkaline phosphatase, *TBIL* Total bilirubin, *DBIL* Direct bilirubin, *hs-CRP* Hypersensitive C-reactive protein

**Table 2:** Multivariate logistic regression analysis of the risk factors for acute pancreatitis after PTBS

	*B*	*P*	*OR*	*95%CI*
Age (< 60 years)	0.94	0.020	2.57	1.16–5.69
Stent across the duodenal papilla	1.87	< 0.001	6.47	2.66–15.70
Visualization of the pancreatic duct	2.73	< 0.001	15.40	6.07–39.03

### Model development

**Fig. 2:**
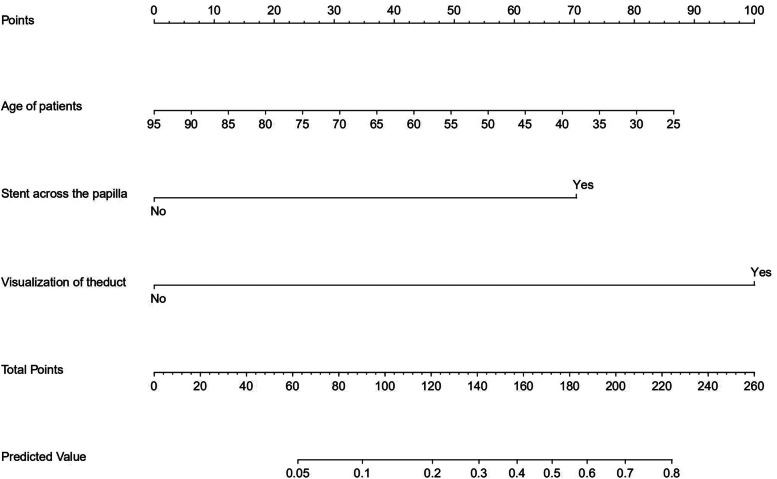
Nomogram for predicting pancreatitis risk. The value of each variable was scored on a point scale from 0 to 100, after which the scores for each variable were added together. That sum is located on the total points axis, which enables us to predict the probability of pancreatitis risk

Based on the multivariate logistic regression analysis, we constructed a nomogram for predicting the probability of post-procedural pancreatitis. In this nomogram, we used the age of patients as a continuous variable, which increases the precision of the model and allows for more individualized risk prediction. Besides, each value of a variable corresponds to a score, and the corresponding scores for the three variables included in the model were summed to achieve a total score for an individual. The total score was then projected onto a total point scale to obtain the probability of post-procedural pancreatitis for each patient (Fig. 2).

### Model validation

**Fig. 3:**
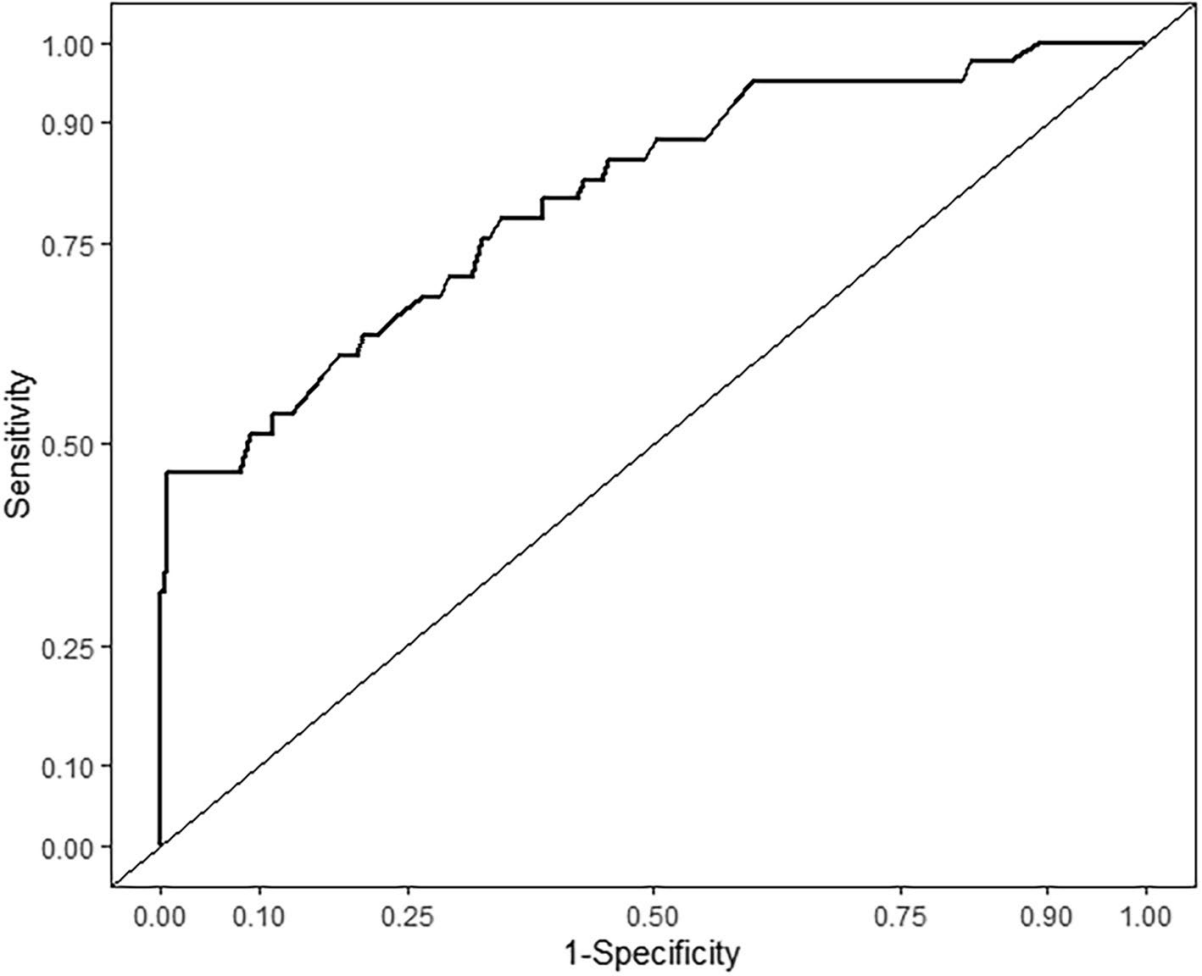
The area under the receiver operator characteristic curve (AUC) of the prediction model: 0.807 with a 95% confidence interval of 0.730 to 0.883

**Fig. 4:**
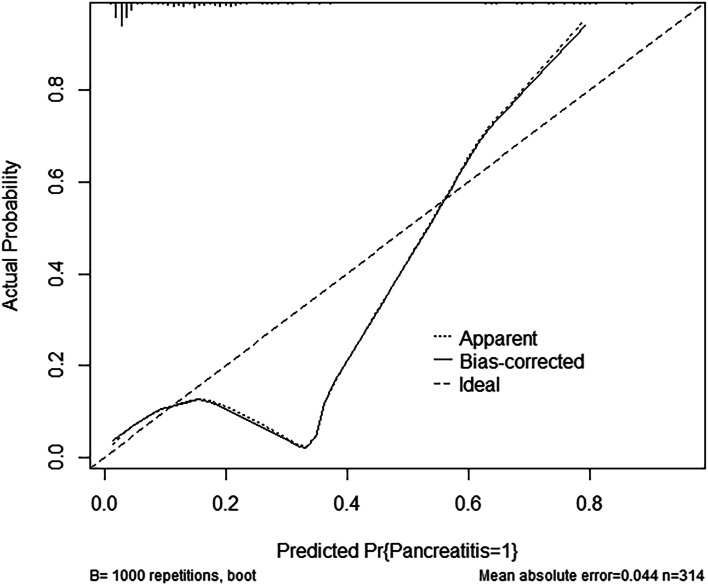
Bootstrap resampling (1000 times) for the prediction model. When the solid line (predicted model) was closer to the dotted line (observed model), the calibration of the model was better

**Fig. 5:**
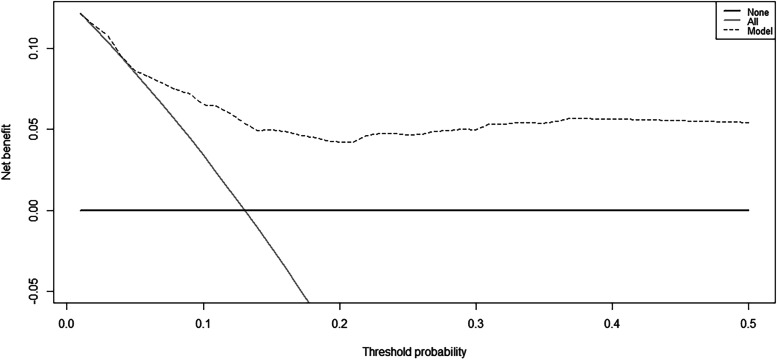
Decision curve analysis (DCA) for the prediction model. The dotted line is for the prediction model, the gray line is for all patients with pancreatitis after PTBS, and the solid horizontal line indicates no patients have pancreatitis. The graph depicts the expected net benefit per patient relative to the model prediction of pancreatitis risk. The net benefit increases as the model curve is extended

The nomogram exhibited great discrimination, with an AUC of 0.807 (*95% CI 0.730 to 0.883*, Fig. 3) and good sensitivity (78.0%) and specificity (65.6%). Second, the optimism-corrected AUC obtained from bootstrap resampling (1000 times) was 0.803, suggesting excellent internal validation. The calibration curve showed that the predicted probabilities of pancreatitis risk agreed well with the observed probabilities (Fig. 4). The DCA curve indicated that the net benefit per patient increases as the model curve is extended, confirming the potential clinical utility of the nomogram (Fig. 5).

## Discussion

In this study, we uncovered that young age, stent insertion across the papilla, and visualization of the pancreatic duct were independent risk factors of pancreatitis following PTBS. Generally, our nomogram incorporating these predictors showed a good predictive ability, which may benefit the clinical management of the procedure.

The results of this study indicated that 41 (13.6%) patients developed pancreatitis after PTBS. Compared with previous endoscopic studies [15–18], the relatively high incidence of post-procedural pancreatitis in this study might be related to several reasons: (1) an excessive contrast agent was injected to evaluate the site and length of obstruction to select a suitable stent, which led to the visualization of the pancreatic duct and subsequent pancreatitis. (2) a high rate (42.0%) of patients who underwent previous biliary drainage including a failed ERCP were enrolled in this study, and secondary treatment may promote the development of pancreatitis.

Previous studies have established that the risk of pancreatitis decreased among elderly patients, which may be due to their low exocrine function and increased tissue fibrosis [16]. In particular, our nomogram uses age as a continuous variable for more individualized risk prediction. After the procedure, a stent across the papilla can contribute significantly to pancreatitis. Tarnasky [17] et al. showed that compression of the pancreatic duct orifice due to the medial defection of biliary stents often results in acute pancreatitis. Notably, during the procedure, visualization of the pancreatic duct exerted a vital role in pancreatitis, which was contained in the model. Freeman [18] et al. demonstrated that the risk of pancreatitis increased among pancreatic duct injection patients. This may result from the high pressure of the pancreatic duct.

Compared with an endoscopic approach, the risk factors of pancreatitis after PTBS remain elusive [7, 9]. Herein, we included more risk factors compared with the previous studies [1, 12], thus developing an effective model, which provides a concrete number and increases the accuracy of prediction. Ideally, the probabilities of pancreatitis per patient can be predicted shortly after the procedure, prophylactic treatment then is used promptly and appropriately to decrease the incidence and prevent negative outcomes.

Nomogram, as a statistical model for individualized predictive of clinical events, was constructed, providing a more intuitive and visual approach to predicting post-procedural pancreatitis in this study. A nomogram has been confirmed can better predict diagnosis, staging, and prognosis in prostate cancer and other diseases than other predictive models such as risk stratification and artificial neural network [19, 20]. We kindly hope this could be practical.

Taken together, demonstration of the pancreatic duct during percutaneous biliary intervention is considered bad practice, thus the contrast medium should be injected carefully with the appropriate pressure. Second, Cosgrove and Zhang [21, 22] et al. reported that stent insertion above the duodenal papilla does not increase the risk of stent occlusion or cholangitis, hence, it might be a more reasonable way of the stent insertion above the papilla if possible. Additionally, patients at high risk of pancreatitis which was identified by the nomogram should be treated with extra measures. Rectal indomethacin may be an alternative postoperative prophylactic treatment, considering several multiple-center prospective trials proposed that indomethacin significantly reduces the incidence of pancreatitis after endoscopic biliary intervention [23, 24]. However, its efficacy on PTBS needs further investigation.

Despite these promising findings, this study has some inherent shortcomings because of its retrospective design. First, data collection was performed retrospectively and this may affect the reliability of the evaluated data. Second, variations in the stent coverings and diameter of the stent were not considered because all stents in this study are uncovered with the same diameter of 8 mm. Given that this was a single-center study, more studies with a larger sample size and involving external validation cohorts are still needed to confirm the present results.

## Conclusion

In the present study, we developed and validated a nomogram to reliably assess the likelihood of pancreatitis after PTBS in patients with MBO, which may enable timely treatment toward high-risk patients and reduce the incidence.

## Data Availability

The datasets generated and/or analysed during the current study are not publicly available, because it is related to subsequent research, but are available from the corresponding author upon reasonable request.

## Abbreviations

PTBS: Percutaneous transhepatic biliary stent insertion
MBO: Malignant biliary obstruction
AUC: Area under the receiver operator characteristic curve
DCA: Decision curve analysis
IQR: Inter-quartile range
